# Shock index and modified shock index at initial clinical assessment in adults with dengue from Oaxaca, Mexico: sex-based differences in a cross-sectional study

**DOI:** 10.3389/fcimb.2026.1864621

**Published:** 2026-10-29

**Authors:** Maricela Martínez Guerra, María Teresa Hernández-Huerta, Eduardo Pérez-Campos, Araceli Guerra Martínez, Jesús Elizarrarás-Rivas, Laura Pérez-Campos Mayoral, Efrén Emmanuel Jarquín González, Héctor Alejandro Cabrera Fuentes, Héctor Martínez-Ruiz

**Affiliations:** 1 Facultad de Medicina y Cirugía, Universidad Autónoma “Benito Juárez” de Oaxaca, Oaxaca, Mexico; 2 Centro de Investigación Facultad de Medicina UNAM-UABJO, Facultad de Medicina y Cirugía, Universidad Autónoma “Benito Juárez” de Oaxaca, Oaxaca, Mexico; 3 SECIHTI, Facultad de Medicina y Cirugía, Universidad Autónoma “Benito Juárez” de Oaxaca, Oaxaca, Mexico; 4 División de Estudios de Posgrado e Investigación, Tecnológico Nacional de México/IT Oaxaca, Oaxaca, Mexico; 5 Coordinación de Investigación en Salud, Instituto Mexicano del Seguro Social (IMSS) Oaxaca, Oaxaca, Mexico; 6 Dirección General de los Servicios de Salud de Oaxaca, Secretaria de Salud, Servicios de Salud de Oaxaca, Oaxaca, Mexico; 7 Hospital General de Zona No.1 Dr. Demetrio Mayoral Pardo, Instituto Mexicano del Seguro Social (IMSS), Oaxaca, Mexico; 8 División de Estudios de Posgrado e Investigación, Tecnológico Nacional de México/Instituto Tecnológico de Tijuana, Tijuana, Mexico; 9 R&D group, Vice Presidency Scientific Research & Innovation, Imam Abdulrahman bin Faisal University (IAU), Dammam, Saudi Arabia

**Keywords:** dengue, hemodynamic assessment, modified shock index, Oaxaca, prognostic nutritional index, sex differences, shock index

## Abstract

**Introduction:**

Dengue-associated plasma leakage and compensatory hemodynamic changes may precede overt hypotension. Evidence on shock index (SI) and modified shock index (MSI) distributions and sex-based differences in adults with dengue remains limited. This study aimed to describe SI and MSI at the initial recorded clinical assessment and compare these indices between women and men. Secondary exploratory analyses examined clinical, laboratory, inflammatory, and immunonutritional variables.

**Methods:**

This cross-sectional observational study included 121 adults with dengue from Oaxaca, Mexico. SI was calculated as heart rate divided by systolic blood pressure, and MSI as heart rate divided by mean arterial pressure. Continuous variables were compared using Welch’s unpaired t-test or the Mann–Whitney U test, as appropriate; categorical variables were compared using Fisher’s exact test. Analyses were descriptive and exploratory.

**Results:**

Participants included 69 women and 52 men; median age was 29.0 years (IQR, 21.0–45.5). Overall median SI was 0.957 (IQR, 0.801–1.137), and mean MSI was 1.257 ± 0.290. Women had higher SI [1.000 (0.811–1.221) versus 0.889 (0.794–1.000); *p* = 0.0122] and MSI (1.318 ± 0.319 versus 1.176 ± 0.225), with a mean MSI difference of 0.142 (95% CI, 0.044–0.240; *p* = 0.0049). Women had higher heart rate, whereas blood pressure measurements did not differ significantly. The neutrophil-to-lymphocyte ratio and systemic immune-inflammation index did not differ significantly by sex. The prognostic nutritional index was lower in women [44.05 (38.43–47.30) versus 47.60 (43.14–53.25); *p* = 0.0007].

**Discussion:**

SI and MSI were higher in women than in men. These differences coincided with higher heart rate, whereas blood pressure measurements did not differ significantly between sexes. Because we did not assess disease severity, diagnostic performance, or longitudinal outcomes, these unadjusted findings are exploratory and require prospective validation before clinical thresholds or prognostic implications can be established.

## Graphical Abstract

**Figure:**
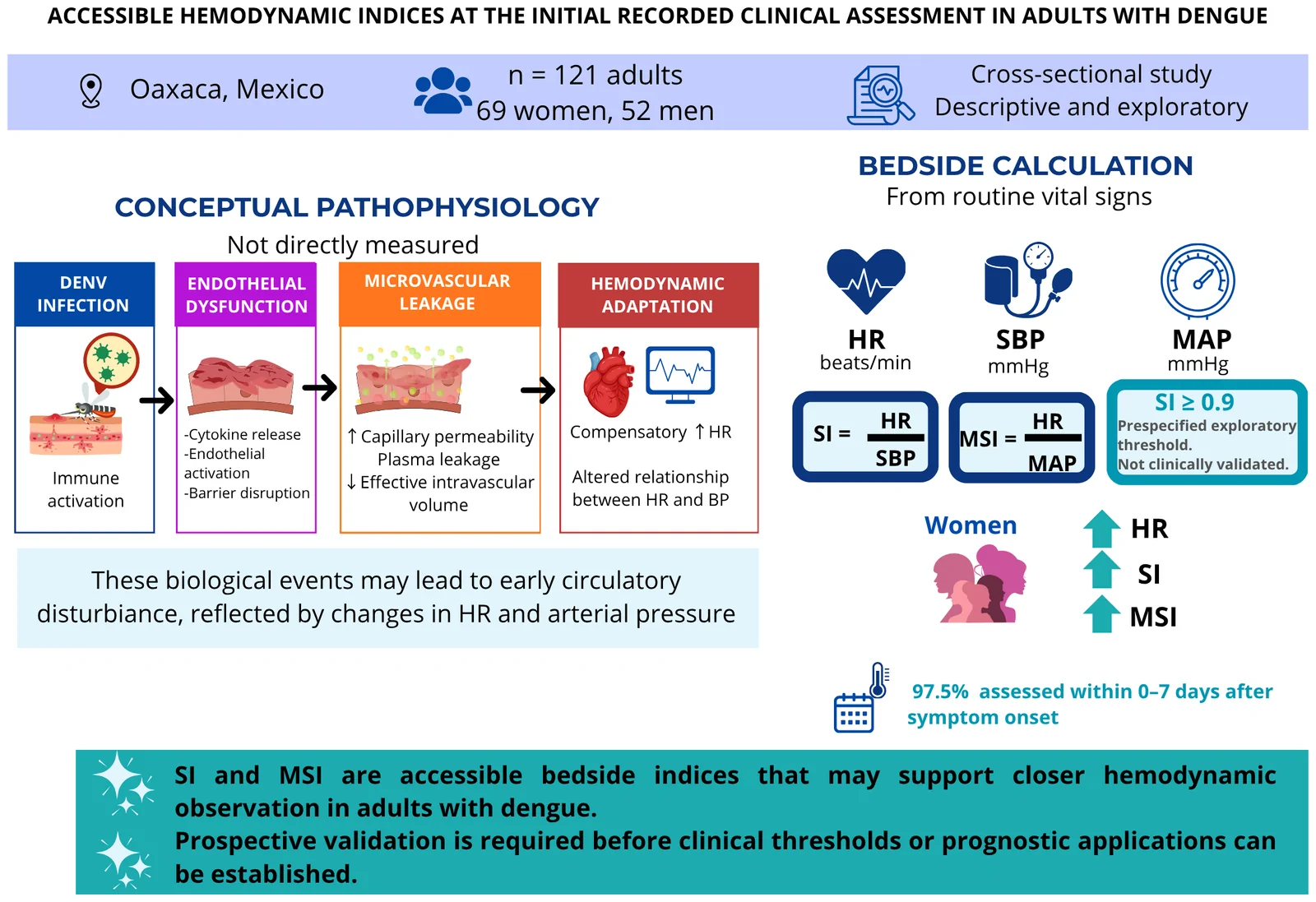
Dengue-associated endothelial dysfunction and plasma leakage may contribute to hemodynamic changes. SI and MSI are accessible indices derived from routine vital signs, and their clinical significance in dengue requires prospective validation. The pathophysiological mechanisms illustrated are presented as a conceptual framework and were not directly assessed in this study. HR, heart rate; MAP, mean arterial pressure; MSI, Modified Shock Index; SI, Shock Index; SBP, systolic blood pressure.

## Highlights

- The overall median Shock Index was close to the prespecified alert threshold at the recorded clinical assessment.
- Shock Index and Modified Shock Index were higher in women than in men.
- Sex-based differences in SI and MSI coincided with a higher heart rate, whereas blood pressure measurements were comparable.
- NLR and SII did not differ significantly by sex, while PNI was lower in women in an exploratory analysis.
- Prospective validation against dengue severity and longitudinal outcomes is required before clinical thresholds can be recommended.

## Introduction

Dengue is a clinically dynamic arboviral infection in which patients may progress from an uncomplicated febrile illness to a critical period characterized by increased vascular permeability, plasma leakage, narrowing pulse pressure, and circulatory shock. The critical phase generally occurs around defervescence and lasts approximately 24–48 hours, rather than corresponding to a fixed interval based exclusively on the number of days after symptom onset (World Health Organization, 2009; McBride et al., 2020; Paz-Bailey et al., 2024; CENAPRECE, 2025). Early recognition of circulatory compromise is especially important because compensatory tachycardia may precede overt hypotension and because access to serial imaging, lactate measurement, or invasive hemodynamic monitoring may be limited in some clinical settings.

The Shock Index (SI), calculated as heart rate divided by systolic blood pressure (SBP), integrates two routinely available vital signs into a single measure of cardiovascular stress. The Modified Shock Index (MSI), calculated as heart rate divided by mean arterial pressure (MAP), also incorporates diastolic pressure and may reflect changes in perfusion pressure not captured by systolic pressure alone (Rady et al., 1994; Koch et al., 2019; Jouffroy et al., 2024). SI and MSI are influenced by age, sex, fever, pain, hydration status, cardiovascular conditions, and medications; therefore, thresholds derived from trauma, sepsis, or obstetric populations cannot be assumed to have identical diagnostic meaning in dengue (Steimer, 2002; Rappaport et al., 2013; Kim et al., 2016; Walter et al., 2016; Koch et al., 2019).

Dengue-specific evidence remains limited but is expanding. In older adults with dengue, SI ≥1 was associated with 30-day mortality, although its sensitivity was low and its specificity was high (Tan et al., 2021). Also, a study of hospitalized adults found that elevated SI and MSI were associated with complicated dengue during hospitalization, supporting further evaluation of these indices in disease-specific populations (Xu et al., 2025). However, their distribution at initial assessment and the extent to which sex-related physiological variation influences their interpretation remain insufficiently described in Latin American adults. Routine hematological and biochemical measurements may provide complementary information regarding the inflammatory and immunonutritional context in which hemodynamic changes occur. Composite indices such as the neutrophil-to-lymphocyte ratio (NLR) (Zahorec, 2001), systemic immune-inflammation index (SII) (Hu et al., 2014), and prognostic nutritional index (PNI) (Onodera et al., 1984; Shabil et al., 2025) can be calculated from routinely available measurements, but their interpretation in acute dengue remains exploratory and may vary by day of illness. In this context, the present study described SI and MSI at the initial recorded clinical assessment and compared these indices between women and men with dengue in Oaxaca, Mexico. Secondary exploratory analyses examined sex-based differences in clinical manifestations, laboratory variables, NLR, SII, and PNI.

## Methods

### Study design and population

This descriptive, exploratory cross-sectional observational study included 121 adults (≥18 years) with RT-qPCR-confirmed dengue from the Central Valleys region of Oaxaca, Mexico. Blood samples were collected from February 9 to October 10, 2024. No formal *a priori* sample-size calculation was performed. A complete screening log was not available for this analysis. Therefore, the total number of individuals assessed for eligibility and the numbers and reasons for exclusion or non-participation before enrollment could not be reported. The final sample size was determined by the number of confirmed adult dengue cases included during this study period. Rapid immunochromatographic testing for NS1 antigen and anti-DENV IgM and IgG antibodies, as well as molecular testing by real-time reverse-transcription polymerase chain reaction (RT-qPCR), was performed by the Oaxaca State Public Health Laboratory as part of routine epidemiological surveillance. RT-qPCR was conducted using the TaqMan™ Arbovirus Triplex RT-qPCR Kit (ZIKV/DENV/CHIKV; Applied Biosystems–Thermo Fisher Scientific), in accordance with the procedures of the Mexican National Network of Public Health Laboratories coordinated by the Instituto de Diagnóstico y Referencia Epidemiológicos (InDRE). The research team received the official qualitative diagnostic results and available serotype information through coded participant identifiers. To preserve patient confidentiality, no directly identifying laboratory information was transferred to the investigators. Raw amplification files, cycle-threshold values, detailed extraction and amplification records, reagent lot numbers, and other internal laboratory metadata were retained by the State Laboratory and were not available to the research team under its institutional data-governance and confidentiality policies. Consequently, the present study reports the verified diagnostic information contained in the official laboratory reports. Rapid NS1/IgM/IgG results were used for complementary descriptive characterization and were not used as the sole criterion for case confirmation.

### Clinical markers of hemodynamic perfusion

Hemodynamic variables included heart rate (HR), systolic blood pressure (SBP), diastolic blood pressure (DBP), mean arterial pressure (MAP), pulse pressure, Shock Index (SI), and Modified Shock Index (MSI). SI was calculated for each participant as heart rate divided by systolic blood pressure: SI = HR/SBP; MAP was calculated from systolic and diastolic blood pressure using the following equation: MAP = (SBP + 2 × DBP)/3; MSI was subsequently calculated as heart rate divided by mean arterial pressure: MSI = HR/MAP; Pulse pressure was calculated as: Pulse pressure = SBP − DBP (Rady et al., 1994; Cecconi et al., 2014; Ochagavía et al., 2014). SI and MSI were derived from the first complete set of contemporaneously recorded vital signs available for each participant at the initial clinical assessment. A single set of HR, SBP, and DBP measurements was used for the analysis; repeated measurements were not averaged. For descriptive purposes, SI ≥0.9 was prespecified as a literature-derived exploratory threshold; it was not evaluated as a diagnostic criterion or used to determine study outcomes. Neurologic status was summarized using the total Glasgow Coma Scale score and categorized as preserved consciousness (GCS 15) or altered mental status (GCS <15) (Jennett, 1997; Palmer and Knight, 2006; Matis and Birbilis, 2008; Muñana-Rodríguez and Ramírez-Elías, 2014). Capillary refill time was recorded in seconds and categorized as normal (≤2 s) or prolonged (>2 s) (Cecconi et al., 2015; Hernández et al., 2018). Serum creatinine (SCR) was analyzed as a continuous laboratory variable related to renal function (Bouglé and Duranteau, 2011; Regueira et al., 2011; Ma et al., 2019). Acute kidney injury was not adjudicated because baseline or serial creatinine measurements and urine-output criteria were unavailable.

Inflammatory and Nutritional Indices were calculated individually for each participant using laboratory measurements obtained from the same blood sample. NLR was calculated as the absolute neutrophil count divided by the absolute lymphocyte count (Zahorec, 2001; Kang et al., 2017). SII was calculated as platelet count × absolute neutrophil count/absolute lymphocyte count (Hu et al., 2014; Chen et al., 2017; Buonacera et al., 2022; Adamescu et al., 2025). The prognostic nutritional index (PNI) was calculated as 10 × serum albumin (g/dL) + 5 × absolute lymphocyte count (×10³/µL) (Onodera et al., 1984; Kang et al., 2017). Participants with missing values for any component required to calculate an index were excluded only from the analysis for which that index was calculated.

### Statistical analysis

Statistical analyses were performed using GraphPad Prism version 5.02 (GraphPad Software, Boston, MA, USA). Analyses were primarily descriptive and exploratory and were conducted for the overall study population and stratified by sex, consistent with the cross-sectional observational design. Continuous variables were summarized as mean ± standard deviation (SD) when their distributions were approximately normal and as median with interquartile range (IQR) when distributions were non-normal. Comparisons between men and women were performed using a two-sided unpaired *t* test with Welch’s correction for normally distributed continuous variables and the two-sided Mann–Whitney *U* test for non-normally distributed variables. When applicable, between-group differences for normally distributed variables were expressed as mean differences (women minus men) with corresponding 95% confidence intervals (CIs). Categorical variables, including clinical manifestations and warning signs, were summarized as absolute frequencies and percentages. For each clinical manifestation, sex-specific proportions and their 95% CIs were calculated using the Wilson score method. Associations between sex and categorical clinical variables were evaluated using the two-sided Fisher’s exact test. Effect estimates were expressed as odds ratios (ORs) with 95% CIs, using men as the reference group; therefore, an OR >1 indicated higher odds of the corresponding manifestation in women, whereas an OR <1 indicated higher odds in men. Variables applicable exclusively to women were analyzed descriptively and excluded from between-sex hypothesis testing. The interval from symptom onset to blood sampling was summarized as absolute frequencies and percentages, without inferential comparisons because few participants were sampled more than seven days after symptom onset. All statistical tests were two-sided, and a *p* value <0.05 was considered statistically significant. Given the exploratory nature of the study, statistical findings were interpreted as hypothesis-generating rather than confirmatory.

### Ethics approval and consent to participate

The study protocol was approved by the Institutional Research Ethics Committee of the Faculty of Medicine and Surgery of Universidad Autónoma “Benito Juárez” de Oaxaca, under approval number 20240207-FMC/UISD. The corresponding institutional and hospital authorizations were obtained before participant recruitment. Written informed consent was obtained from all participants or their representatives prior to inclusion.

## Results

At the time of blood sampling, 118/121 participants (97.5%) were assessed within 0–7 days after symptom onset, including 68/69 women (98.6%) and 50/52 men (96.2%) (**Table 1**). Three participants (2.5%) were assessed 8–9 days after symptom onset, comprising one woman and two men. No participants were assessed 10–14 days after symptom onset. Rapid dengue diagnostic marker results were available for all 121 participants. Diagnostic testing was performed by the Oaxaca State Public Health Laboratory, and the research team received the corresponding official results through coded identifiers. DENV infection was confirmed by RT-qPCR, and DENV-3 was reported as the circulating serotype. Rapid NS1 antigen and anti-DENV IgM and IgG results were available for all participants and were analyzed as complementary descriptive markers. Raw RT-qPCR amplification data and cycle-threshold values were not available to the investigators. NS1 antigen was reactive in 116/121 participants (95.9%), including 65/69 women (94.2%) and 51/52 men (98.1%), without a statistically significant sex-based difference [OR = 0.32 (95% CI, 0.03–2.94); Fisher’s exact p = 0.390]. IgG was reactive in 23/121 participants (19.0%), comprising 17/69 women (24.6%) and 6/52 men (11.5%) [OR = 2.51 (95% CI, 0.91–6.90); p = 0.101]. IgM was reactive in 12/121 participants (9.9%), including 11/69 women (15.9%) and 1/52 man (1.9%) [OR = 9.67 (95% CI, 1.21–77.57); nominal p = 0.012]. Although IgM reactivity was more frequent in women, the estimate was based on a small number of reactive cases and had a wide confidence interval; therefore, it should be interpreted as exploratory. The distribution of clinical manifestations according to sex is shown in **Table 2**. Headache, myalgia, nausea, arthralgia, anorexia, and lethargy were among the most frequently observed manifestations in both women and men. Headache was reported in 95.7% of women and 94.2% of men, myalgia in 91.3% and 92.3%, nausea in 89.9% and 92.3%, and arthralgia in 91.3% and 88.5%, respectively. Most clinical manifestations showed no statistically significant differences between sexes. However, diarrhea was significantly more frequent in men than in women [59.6% vs. 39.1%; OR = 0.44 (95% CI, 0.21–0.91); *p* = 0.029]. Similarly, Islands of white in a sea of red were markedly more frequent in men [40.4% vs. 11.6%; OR = 0.19 (95% CI, 0.08–0.49); *p* = 0.0004]. In contrast, lipothymy was substantially more frequent in women than in men [23.2% vs. 1.9%; OR = 15.40 (95% CI, 1.97–120.40); *p* = 0.0009]. Manifestations suggestive of plasma leakage were uncommon in both sexes. Ascites was observed in 1.4% of women and 1.9% of men; pleural effusion in 2.9% of women and 1.9% of men; and pericardial effusion in 0.0% of women and 1.9% of men, with no significant sex-related differences. Hemorrhagic manifestations such as hematemesis and melena were also relatively infrequent and did not differ significantly between women and men. Heavy metrorrhagia was documented in 5/69 women (7.2%; 95% CI, 3.1–15.9%) and was considered not applicable to men.

**Table 1:** Interval from symptom onset to blood sampling and rapid dengue diagnostic marker reactivity in the study population.

Interval from symptom onset to blood sampling					
Days		Total, n/N (%)	Women, n/N (%)	Men, n/N (%)	
0–7		118/121 (97.5)	68/69 (98.6)	50/52 (96.2)	
8–9		3/121 (2.5)	1/69 (1.4)	2/52 (3.8)	
10–14		0/121 (0.0)	0/69 (0.0)	0/52 (0.0)	

Rapid diagnostic marker results					
Rapid diagnostic marker	Total, n/N (%)	Women, n/N (%)	Men, n/N (%)	OR (95% CI)	*p* value
NS1 antigen reactive	116/121 (95.9)	65/69 (94.2)	51/52 (98.1)	0.32 (0.03–2.94)	0.390
IgM reactive	12/121 (9.9)	11/69 (15.9)	1/52 (1.9)	9.67 (1.21–77.57)	0.012
IgG reactive	23/121 (19.0)	17/69 (24.6)	6/52 (11.5)	2.51 (0.91–6.90)	0.101

Combined rapid diagnostic marker patterns					
Combined rapid diagnostic pattern		Total, n/N (%)	Women, n/N (%)	Men, n/N (%)	
NS1+/IgM−/IgG−		97/121 (80.2)	52/69 (75.4)	45/52 (86.5)	
NS1+/IgM−/IgG+		10/121 (8.3)	5/69 (7.2)	5/52 (9.6)	
NS1+/IgM+/IgG+		8/121 (6.6)	8/69 (11.6)	0/52 (0.0)	
NS1+/IgM+/IgG−		1/121 (0.8)	0/69 (0.0)	1/52 (1.9)	
NS1−/IgM+/IgG+		3/121 (2.5)	3/69 (4.3)	0/52 (0.0)	
NS1−/IgM−/IgG+		2/121 (1.7)	1/69 (1.4)	1/52 (1.9)	

Data are presented as n/N (%). Intervals represent the time from symptom onset to blood sampling; febrile, critical, and recovery phases were not assigned because the timing of defervescence was not systematically documented. No participants were assessed 10–14 days after symptom onset. Combined marker patterns were mutually exclusive, and each participant was included in one category. Patterns with zero observations (NS1−/IgM+/IgG− and NS1−/IgM−/IgG−) are not shown. Individual marker rows were not mutually exclusive because participants could have more than one reactive marker. Between-sex comparisons of individual marker reactivity were performed using the two-sided Fisher’s exact test. Odds ratios compare women with men, using men as the reference group. Combined patterns were summarized descriptively because of sparse or zero cell counts. P values are nominal and should be interpreted as exploratory. Percentages may not total 100% because of rounding. CI, confidence interval; IgG, immunoglobulin G; IgM, immunoglobulin M; NS1, nonstructural protein 1; OR, odds ratio.

**Table 2:** Sex-specific distribution of clinical manifestations, warning signs, and bedside perfusion findings among adults with dengue.

Clinical manifestation	Women, n/N (%) [95% CI]	Men, n/N (%) [95% CI]	OR (95% CI)	*p*
Conjunctivitis	25/69 (36.2) [25.9–48.0]	24/52 (46.2) [33.3–59.5]	0.66 (0.32–1.38)	0.350
Anorexia	51/69 (73.9) [62.5–82.8]	40/52 (76.9) [63.9–86.3]	0.85 (0.37–1.97)	0.832
Diarrhea	27/69 (39.1) [28.5–50.9]	31/52 (59.6) [46.1–71.8]	0.44 (0.21–0.91)	**0.029**
Pharyngeal hyperemia	25/69 (36.2) [25.9–48.0]	18/52 (34.6) [23.2–48.2]	1.07 (0.51–2.28)	1.000
Pruritus	28/69 (40.6) [29.8–52.4]	26/52 (50.0) [36.9–63.1]	0.68 (0.33–1.41)	0.357
Joint edema	20/69 (29.0) [19.6–40.6]	12/52 (23.1) [13.7–36.1]	1.36 (0.59–3.12)	0.535
Diaphoresis	43/69 (62.3) [50.5–72.8]	33/52 (63.5) [49.9–75.2]	0.95 (0.45–2.01)	1.000
Nausea	62/69 (89.9) [80.5–95.0]	48/52 (92.3) [81.8–97.0]	0.74 (0.20–2.67)	0.756
Exanthema	31/69 (44.9) [33.8–56.6]	32/52 (61.5) [48.0–73.5]	0.51 (0.25–1.06)	0.098
Myalgia	63/69 (91.3) [82.3–96.0]	48/52 (92.3) [81.8–97.0]	0.88 (0.23–3.28)	1.000
Arthralgia	63/69 (91.3) [82.3–96.0]	46/52 (88.5) [77.0–94.6]	1.37 (0.42–4.52)	0.760
Headache	66/69 (95.7) [88.0–98.5]	49/52 (94.2) [84.4–98.0]	1.35 (0.26–6.96)	1.000
Retro-orbital pain	41/69 (59.4) [47.6–70.2]	28/52 (53.8) [40.5–66.7]	1.26 (0.61–2.60)	0.581
Petechiae	21/69 (30.4) [20.8–42.1]	13/52 (25.0) [15.2–38.2]	1.31 (0.58–2.95)	0.546
Gingival bleeding	15/69 (21.7) [13.6–32.8]	13/52 (25.0) [15.2–38.2]	0.83 (0.36–1.95)	0.671
Epistaxis	20/69 (29.0) [19.6–40.6]	17/52 (32.7) [21.5–46.2]	0.84 (0.39–1.83)	0.694
Positive Rumpel-Leede test	16/69 (23.2) [14.8–34.4]	12/52 (23.1) [13.7–36.1]	1.01 (0.43–2.36)	1.000
Islands of white in a sea of red	8/69 (11.6) [6.0–21.2]	21/52 (40.4) [28.2–53.9]	0.19 (0.08–0.49)	**0.0004**
Severe abdominal pain	37/69 (53.6) [42.0–64.9]	24/52 (46.2) [33.3–59.5]	1.35 (0.66–2.78)	0.465
Ascites	1/69 (1.4) [0.3–7.8]	1/52 (1.9) [0.3–10.1]	0.75 (0.05–12.29)	1.000
Pleural effusion	2/69 (2.9) [0.8–10.0]	1/52 (1.9) [0.3–10.1]	1.52 (0.13–17.27)	1.000
Pericardial effusion	0/69 (0.0) [0.0–5.3]	1/52 (1.9) [0.3–10.1]	0.25 (0.01–6.19)	0.430
Swelling/edema	4/69 (5.8) [2.3–14.0]	3/52 (5.8) [2.0–15.6]	1.01 (0.21–4.70)	1.000
Lymphadenopathy	2/69 (2.9) [0.8–10.0]	5/52 (9.6) [4.2–20.6]	0.28 (0.05–1.51)	0.138
Weight loss	19/69 (27.5) [18.4–39.0]	16/52 (30.8) [19.9–44.3]	0.86 (0.39–1.89)	0.840
Lethargy	56/69 (81.2) [70.4–88.6]	36/52 (69.2) [55.7–80.1]	1.92 (0.82–4.45)	0.139
Postural hypotension	43/69 (62.3) [50.5–72.8]	27/52 (51.9) [38.7–64.9]	1.53 (0.74–3.18)	0.270
Lipothymy	16/69 (23.2) [14.8–34.4]	1/52 (1.9) [0.3–10.1]	15.40 (1.97–120.40)	**0.0009**
Hepatomegaly >2 cm	6/69 (8.7) [4.0–17.7]	4/52 (7.7) [3.0–18.2]	1.14 (0.31–4.28)	1.000
Cold extremities	14/69 (20.3) [12.5–31.2]	6/52 (11.5) [5.4–23.0]	1.95 (0.69–5.49)	0.226
Undetectable pulse	9/69 (13.0) [7.0–23.0]	3/52 (5.8) [2.0–15.6]	2.45 (0.63–9.55)	0.230
Hematemesis	3/69 (4.3) [1.5–12.0]	5/52 (9.6) [4.2–20.6]	0.43 (0.10–1.88)	0.287
Melena (black and tarry stools)	6/69 (8.7) [4.0–17.7]	3/52 (5.8) [2.0–15.6]	1.56 (0.37–6.54)	0.731
Heavy metrorrhagia	5/69 (7.2) [3.1–15.9]	N/A	—	—
Glasgow Coma Scale <15	3/69 (4.3) [1.5–12.0]	1/52 (1.9) [0.3–10.1]	2.32 (0.23–22.96)	0.634
Prolonged capillary refill time (>2 s)	16/69 (23.2) [14.8–34.4]	6/52 (11.5) [5.4–23.0]	2.31 (0.84–6.41)	0.152

Data are presented as n/N (%) [95% CI]. *n* represents the number of participants presenting the clinical manifestation and *N* the number of participants evaluated. The 95% confidence intervals for proportions were calculated using the Wilson method. Odds ratios (ORs) compare women with men; therefore, an OR >1 indicates higher odds of the manifestation in women, whereas an OR <1 indicates higher odds in men. Between-sex comparisons were performed using the two-sided Fisher’s exact test. Statistical significance was defined as *p* < 0.05. Heavy metrorrhagia was evaluated only in women and was considered not applicable (N/A) to men; therefore, no OR or *p* value was calculated for this variable. GCS <15 was categorized as altered neurologic status, with GCS 15 as the reference category. Capillary refill time >2 s was categorized as prolonged, with capillary refill time ≤2 s as the reference category. CI, confidence interval; OR, odds ratio; N/A, not applicable. Bold p values indicate nominal statistical significance (two-sided p < 0.05). Findings should be interpreted as exploratory.

The clinical and hemodynamic characteristics are shown in **Table 3**. The median age of the study population was 29.0 years (IQR, 21.0–45.5). Age, body mass index, and waist-to-hip ratio were comparable between men and women, with no statistically significant differences between groups. In the overall cohort, SI values ranged from 0.438 to 1.614, with a mean ± SD of 0.982 ± 0.241 and a median (IQR) of 0.957 (0.801–1.137). MSI values ranged from 0.656 to 1.935, with a mean ± SD of 1.257 ± 0.290 and a median (IQR) of 1.212 (1.036–1.479). Women had higher SI values than men [median (IQR), 1.000 (0.811–1.221) vs. 0.889 (0.794–1.000), respectively; Mann–Whitney U test, p = 0.0122]. Similarly, MSI was higher in women than in men [mean ± SD, 1.318 ± 0.319 vs. 1.176 ± 0.225, respectively], with a mean difference of 0.142 (95% CI, 0.044–0.240; Welch’s unpaired t test, p = 0.0049). 73/121 participants (60.3%) had SI ≥0.9 and 52/121 (43.0%) had SI ≥1.0. Among women, the corresponding proportions were 48/69 (69.6%) and 38/69 (55.1%), whereas among men they were 25/52 (48.1%) and 14/52 (26.9%), respectively. These threshold-based proportions were summarized descriptively and were not evaluated for sensitivity, specificity, diagnostic discrimination, disease severity, or prognostic performance.

**Table 3:** Demographic, anthropometric, and hemodynamic characteristics of adult patients with dengue, overall and stratified by sex.

Variable	Total (n = 121)	Men (n = 52)	Women (n = 69)	p value
Demographic variables				
Age, years	29.0 (21.0–45.5)	29.0 (20.0–39.75)	29.0 (23.0–51.50)	0.3103
Sex, %	100	43.0	57.0	—
Anthropometric characteristics				
Body mass index, kg/m²	25.67 (22.56–29.49)	25.49 (21.98–29.38)	27.06 (23.04–29.85)	0.3934
Waist-to-hip ratio	0.900 (0.894–1.000)	0.900 (0.893–1.000)	0.900 (0.893–0.900)	0.1536
Hemodynamic window				
Shock Index				
Mean ± SD	0.982 ± 0.241	0.917 ± 0.204	1.031 ± 0.256	—
Median (IQR)	0.957 (0.801–1.137)	0.889 (0.794–1.000)	1.000 (0.811–1.221)	0.0122^a^
Minimum–maximum	0.438–1.614	0.438–1.614	0.517–1.613	—
SI ≥0.9, n/N (%)	73/121 (60.3)	25/52 (48.1)	48/69 (69.6)	—
SI ≥1.0, n/N (%)	52/121 (43.0)	14/52 (26.9)	38/69 (55.1)	—
Modified Shock Index				
Mean ± SD	1.257 ± 0.290	1.176 ± 0.225	1.318 ± 0.319	0.0049^b^
Median (IQR)	1.212 (1.036–1.479)	1.143 (1.064–1.256)	1.286 (1.021–1.567)	—
Minimum–maximum	0.656–1.935	0.656–1.784	0.675–1.935	—
Heart rate, beats/min	95.41 ± 19.40	91.42 ± 15.94	98.42 ± 21.26	0.0407
Systolic blood pressure, mmHg	100 (90–110)	100 (90–110)	100 (90–110)	0.1836
Diastolic blood pressure, mmHg	60 (60–70)	61 (60–70)	60 (60–70)	0.4116
Pulse pressure, mmHg	30 (29–40)	30 (30–40)	30 (20–40)	0.2145
Mean arterial pressure, mmHg	76.7 (70.0–83.3)	76.7 (70.0–86.7)	73.3 (70.0–83.3)	0.1949

Data are presented as mean ± standard deviation (SD), median with interquartile range (IQR), minimum–maximum, or n/N (%), as indicated. For SI and MSI, both parametric and nonparametric descriptive statistics are reported to provide a complete characterization of their distributions. ^a^The p value for SI corresponds to the two-sided Mann–Whitney U test comparing the complete distributions between women and men. ^b^The p value for MSI corresponds to Welch’s two-sided unpaired t test. —, not applicable or not estimated; IQR, interquartile range; MSI, Modified Shock Index; SD, standard deviation; SI, Shock Index.

Women also had a higher heart rate than men (98.42 ± 21.26 vs. 91.42 ± 15.94 beats/min), corresponding to a mean difference of 7.00 beats/min (95% CI, 0.30–13.69; *p* = 0.0407). In contrast, systolic blood pressure, diastolic blood pressure, pulse pressure, and mean arterial pressure did not differ significantly between sexes.

Baseline hematological and coagulation characteristics, as well as renal, protein, and enzyme-related laboratory characteristics, are presented in **Table 4**. The median platelet count was 78.0 × 10³/µL (IQR, 37.5–143.0), and the median total leukocyte count was 4.37 × 10³/µL (IQR, 3.31–6.52). Men had higher hemoglobin concentrations than women [16.40 (15.40–17.00) vs. 14.30 (13.00–15.30) g/dL; *p* < 0.0001] and a higher hematocrit [47.35% (44.55–49.83) vs. 42.20% (38.30–45.05); *p* < 0.0001]. The absolute monocyte count was also higher in men than in women [0.510 (0.373–1.000) vs. 0.420 (0.265–0.530) × 10³/µL; *p* = 0.0031]. No statistically significant differences were found in mean corpuscular volume, mean corpuscular hemoglobin, platelet count, total leukocyte count, absolute neutrophil count, neutrophil percentage, absolute lymphocyte count, or absolute basophil count. Coagulation parameters were comparable between sexes. No significant differences were observed in prothrombin time, international normalized ratio, activated partial thromboplastin time, or fibrinogen concentration. C-reactive protein and glucose concentrations were also similar between men and women.

**Table 4:** Baseline hematological, hemostatic, and biochemical characteristics of adult patients with dengue, overall and stratified by sex.

Variable	Total (n = 121)	Men (n = 52)	Women (n = 69)	p value
Hematological parameters				
Hemoglobin, g/dL	15.30 (14.20–16.40)	16.40 (15.40–17.00)	14.30 (13.00–15.30)	<0.0001
Hematocrit, %	44.40 (40.90–47.85)	47.35 (44.55–49.83)	42.20 (38.30–45.05)	<0.0001
Mean corpuscular volume, fL	86.30 (83.00–88.85)	86.15 (83.58–88.93)	86.60 (82.40–88.85)	0.5523
Mean corpuscular hemoglobin, pg	29.30 (28.20–30.50)	29.30 (28.73–30.78)	29.20 (27.90–30.30)	0.1483
Platelet count, ×10³/µL	78.0 (37.5–143.0)	81.0 (33.75–143.5)	74.0 (37.50–143.0)	0.9562
Total leukocyte count, ×10³/µL	4.37 (3.31–6.52)	4.56 (3.46–6.55)	4.07 (3.07–6.52)	0.2275
Absolute neutrophil count, ×10³/µL	2.30 (1.44–3.43)	2.37 (1.49–3.33)	2.30 (1.39–3.64)	0.9000
Neutrophils, %	51.20 (34.85–69.35)	50.15 (35.63–60.18)	55.20 (34.45–72.00)	0.1224
Absolute lymphocyte count, ×10³/µL	1.30 (0.82–2.24)	1.54 (1.18–2.74)	1.24 (0.78–2.12)	0.0676
Absolute monocyte count, ×10³/µL	0.460 (0.300–0.690)	0.510 (0.373–1.000)	0.420 (0.265–0.530)	0.0031
Absolute basophil count, ×10³/µL	0.030 (0.015–0.050)	0.030 (0.020–0.060)	0.020 (0.010–0.045)	0.1706
Hemostatic and coagulation parameters				
Prothrombin time (PT), s	13.60 (13.00–14.60)	13.30 (12.65–14.20)	13.80 (13.10–14.75)	0.0531
INR	1.00 (1.00–1.10)	1.00 (1.00–1.10)	1.10 (1.00–1.10)	0.0573
Activated partial thromboplastin time (aPTT), s	32.80 (29.40–37.35)	33.55 (29.40–38.23)	32.60 (29.55–36.75)	0.8423
Fibrinogen, mg/dL	308.0 (265.5–366.5)	313.5 (278.5–366.3)	303.0 (253.0–366.5)	0.7414
Inflammatory and metabolic parameters				
C-reactive protein (CRP), mg/dL	1.00 (0.80–1.80)	1.00 (0.80–1.78)	1.00 (0.80–1.90)	0.9454
Glucose, mg/dL	100.0 (89.0–116.5)	100.0 (89.25–114.0)	100.0 (88.50–122.5)	0.9582
Renal function parameters				
Creatinine, mg/dL	0.80 (0.60–0.92)	0.90 (0.80–1.02)	0.60 (0.50–0.80)	<0.0001
Blood urea nitrogen (BUN), mg/dL	11.0 (8.0–15.5)	12.0 (10.0–19.0)	10.0 (7.0–14.0)	0.0120
Estimated glomerular filtration rate (eGFR), mL/min/1.73 m²	107.0 (80.5–123.5)	88.5 (75.5–108.8)	116.0 (97.0–131.0)	<0.0001
Urea, mg/dL	23.54 (17.06–32.10)	25.68 (21.40–39.63)	21.40 (14.98–32.10)	0.0170
Serum protein profile				
Albumin, g/dL	3.80 (3.40–4.10)	3.90 (3.60–4.20)	3.70 (3.15–4.00)	0.0091
Globulin, g/dL	3.22 (3.00–3.60)	3.20 (3.03–3.58)	3.30 (3.00–3.60)	0.9895
Albumin-to-globulin ratio (A/G ratio)	1.10 (1.00–1.25)	1.20 (1.10–1.30)	1.10 (0.95–1.20)	0.0129
Hepatic and hepatobiliary parameters				
Aspartate aminotransferase (AST), U/L	190.0 (76.5–302.0)	180.0 (71.5–275.5)	200.0 (79.0–313.0)	0.5332
Alanine aminotransferase (ALT), U/L	94.0 (51.5–191.0)	99.0 (58.0–165.5)	88.0 (50.0–217.0)	0.7375
ALT/AST	0.631 (0.400–0.900)	0.70 (0.50–0.98)	0.60 (0.40–0.80)	0.0359
Alkaline phosphatase (ALP), U/L	97.0 (79.0–131.5)	99.15 (83.75–122.8)	96.00 (76.50–160.5)	0.5027
Gamma-glutamyl transferase (GGT), U/L	61.0 (26.0–185.0)	74.0 (40.0–198.0)	54.0 (21.5–166.5)	0.2368
Tissue injury enzymes				
Creatine kinase (CK), U/L	75.0 (49.0–145.5)	86.0 (56.75–178.0)	70.0 (49.0–100.0)	0.0453
Lactate dehydrogenase (LDH), U/L	321.0 (177.5–658.0)	297.0 (149.3–862.0)	323.0 (183.0–599.0)	0.9165
Inflammatory and immunonutritional composite indices				
NLR	1.46 (0.72–3.33)	1.37 (0.77–2.10)	1.92 (0.72–4.06)	0.097
SII, ×10^9^/L	94.54 (30.72–385.0)	77.1 (32.0–216.1)	137.0 (29.2–511.0)	0.213
PNI	45.20 (40.38–49.85)	47.60 (43.14–53.25)	44.05 (38.43–47.30)	**0.0007**

Data are presented as mean ± standard deviation (SD) or median and interquartile range (IQR), as appropriate. *p* values correspond to two-sided comparisons between men and women. Normally distributed continuous variables were compared using an unpaired *t* test with Welch’s correction, whereas non-normally distributed variables were compared using the Mann–Whitney *U* test. Statistical significance was defined as *p* < 0.05. A/G, albumin-to-globulin ratio; ALP, alkaline phosphatase; ALT, alanine aminotransferase; aPTT, activated partial thromboplastin time; AST, aspartate aminotransferase; BUN, blood urea nitrogen; CK, creatine kinase; CRP, C-reactive protein; eGFR, estimated glomerular filtration rate; GGT, gamma-glutamyl transferase; INR, international normalized ratio; IQR, interquartile range; LDH, lactate dehydrogenase; PT, prothrombin time; SD, standard deviation; NLR, neutrophil-to-lymphocyte ratio; SII, systemic immune-inflammation index; PNI, prognostic nutritional index. Bold p values indicate nominal statistical significance (two-sided p < 0.05). Findings should be interpreted as exploratory.

Sex-related differences were observed in several renal-associated laboratory variables. Men had higher creatinine concentrations than women [0.90 (0.80–1.02) vs. 0.60 (0.50–0.80) mg/dL; *p* < 0.0001], as well as higher blood urea nitrogen [12.0 (10.0–19.0) vs. 10.0 (7.0–14.0) mg/dL; *p* = 0.0120] and urea concentrations [25.68 (21.40–39.63) vs. 21.40 (14.98–32.10) mg/dL; *p* = 0.0170]. Conversely, the estimated glomerular filtration rate was higher in women than in men [116.0 (97.0–131.0) vs. 88.5 (75.5–108.8) mL/min/1.73 m²; *p* < 0.0001]. Men also had higher albumin concentrations [3.90 (3.60–4.20) vs. 3.70 (3.15–4.00) g/dL; *p* = 0.0091] and a higher albumin-to-globulin ratio [1.20 (1.10–1.30) vs. 1.10 (0.95–1.20); *p* = 0.0129]. Globulin concentrations did not differ between groups. Regarding enzyme-related variables, the ALT/AST ratio was higher in men than in women [0.70 (0.50–0.98) vs. 0.60 (0.40–0.80); *p* = 0.0359]. Creatine kinase activity was also higher in men [86.0 (56.75–178.0) vs. 70.0 (49.0–100.0) U/L; *p* = 0.0453]. No statistically significant differences were observed in aspartate aminotransferase, alanine aminotransferase, alkaline phosphatase, gamma-glutamyl transferase, or lactate dehydrogenase.

Inflammatory and nutritional indices were analyzed as non-normally distributed variables and are therefore presented as median (IQR), **Table 4**. The NLR was numerically higher in women than in men [1.92 (0.72–4.06) vs. 1.37 (0.77–2.10), respectively], although the difference was not statistically significant (Mann–Whitney *U* = 1477, *p* = 0.097). The SII was higher in women [137.0 (29.2–511.0)] than in men [77.1 (32.0–216.1)], but this difference did not reach statistical significance (*U* = 1558, *p* = 0.213). In contrast, the PNI was significantly lower in women than in men [44.05 (38.43–47.30) vs. 47.60 (43.14–53.25), respectively; *U* = 1150, *p* = 0.0007]. These findings indicate that, whereas systemic inflammatory indices were broadly comparable between sexes, women exhibited a significantly lower composite nutritional–immune index.

## Discussion

The principal cross-sectional finding was that SI and MSI values at the initial recorded assessment were higher in women than in men, as were heart rates, with no statistically significant differences in arterial pressure. These findings characterize sex-stratified distributions of the indices but do not establish early circulatory disturbance, diagnostic accuracy, or prognostic utility. NLR, SII, and PNI were secondary exploratory analyses. Median SI was 0.957, close to the prespecified alert threshold of 0.9, whereas the mean MSI was 1.257. Women also had a higher heart rate, while systolic blood pressure, diastolic blood pressure, pulse pressure, and mean arterial pressure did not differ significantly between sexes. In secondary analyses, NLR and SII showed no statistically significant sex-based differences, whereas PNI was lower in women. These results represent unadjusted cross-sectional associations and should be interpreted as descriptive and hypothesis-generating. **Figure 1** summarizes the conceptual pathophysiological framework, bedside calculation of SI and MSI, principal observed hemodynamic findings, and their clinical interpretation.

**Figure 1:**
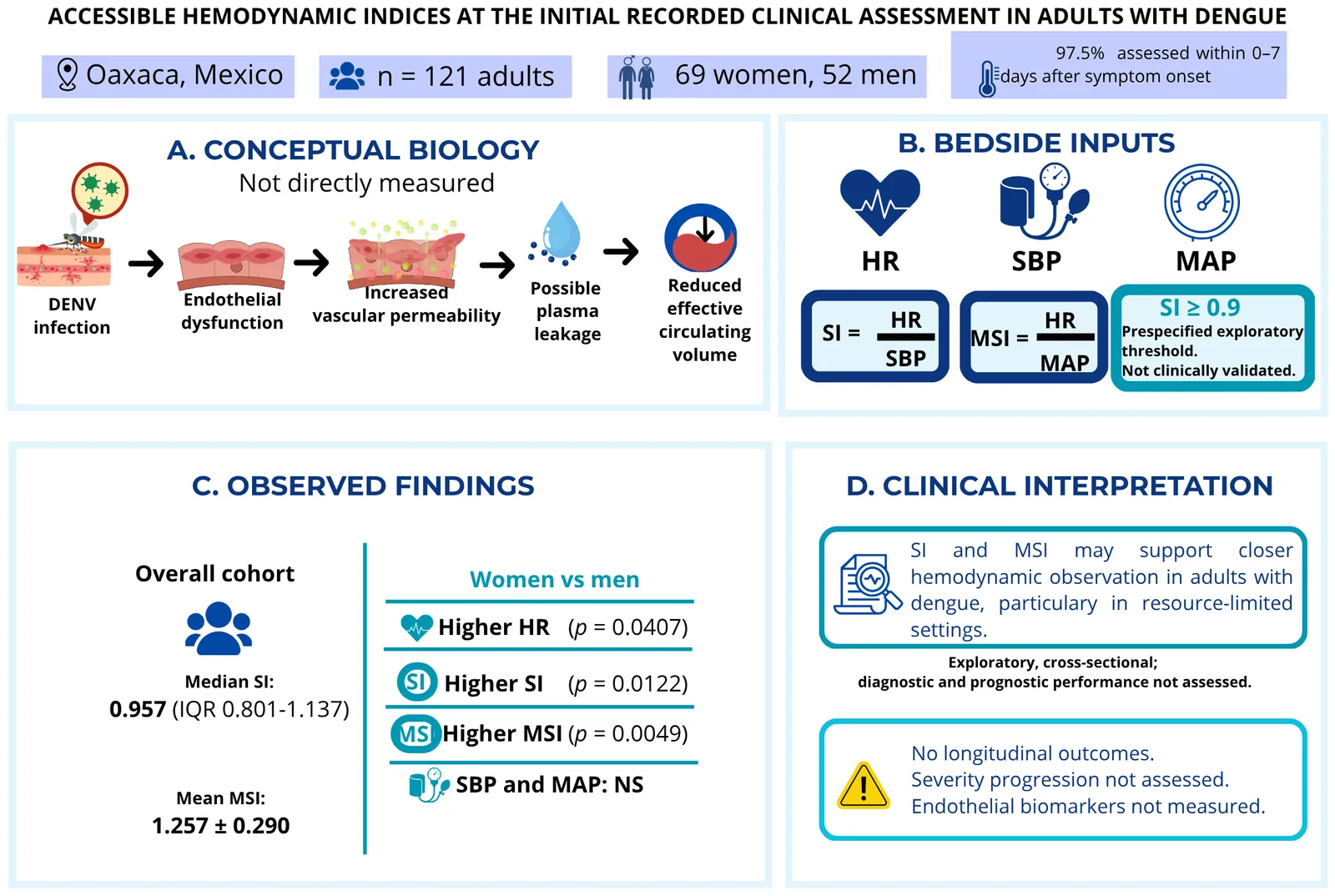
Conceptual pathophysiological framework and observed bedside hemodynamic findings in adults with dengue. DENV, dengue virus; HR, heart rate; MAP, mean arterial pressure; MSI, Modified Shock Index; SI, Shock Index; NS, not statistically significant; SBP, systolic blood pressure.

SI distribution suggests that a substantial proportion of this cohort may have had increased cardiovascular stress at the time of assessment. However, a group median close to or above a selected threshold cannot, by itself, establish early circulatory disturbance in individual patients. The threshold of 0.9 has been used predominantly in emergency, trauma, hemorrhagic, and other acute-care populations, and its diagnostic meaning is influenced by population characteristics and clinical context (Rady et al., 1994; Steimer, 2002; Koch et al., 2019). In adults with dengue, Tan et al. found that SI ≥1 had high specificity but low sensitivity for 30-day mortality in an older population (Tan et al., 2021). A more recent study reported that elevated SI and MSI were associated with complicated dengue during hospitalization, although the study population, thresholds, and clinical outcome differed from those of the present investigation (Xu et al., 2025). These results also align with a recent publication covering 146 dengue prediction studies, which identified shock as one of the predictors most consistently associated with adverse outcomes and incorporated evidence from adults linking elevated SI and MSI values ​​to complicated dengue. However, the review also revealed that the majority of available prediction studies carried a high risk of bias and that external validation was infrequent. Consequently, the available evidence supports the need for further evaluation of hemodynamic indices in dengue but does not establish universal thresholds or direct applicability across different populations (Delpino et al., 2026). Thus, our results complement existing evidence on dengue by describing the distribution of these indices in a younger Mexican cohort, but they do not establish their diagnostic or prognostic performance.

We observed that sex-based variation is particularly relevant for interpretation. Both SI and MSI values were higher in women, and the analysis suggests this was primarily due to higher heart rate rather than to a statistically significant reduction in systolic or mean arterial pressure. Previous population-based data have shown that SI distributions vary by age and sex, generally being higher in women across all age groups (Rappaport et al., 2013). Consequently, using a single universal SI threshold may lead to different classifications of physiological and pathological variation between women and men. Fever, pain, anxiety, hydration status, medication, pregnancy status, and prehospital fluid administration can also alter heart rate and blood pressure (Steimer, 2002; Walter et al., 2016; Koch et al., 2019). Because these variables were not included in an adjusted model, the present study cannot determine whether sex was independently associated with SI or MSI. Furthermore, the lower p-value observed for MSI does not demonstrate that it is more sensitive than SI; such a conclusion would require a direct comparison of discrimination capacity against a predefined clinical outcome.

No statistically significant difference was detected between sexes in NLR and SII. These indices are mathematically dependent on circulating neutrophil, lymphocyte, and platelet counts and may vary substantially throughout the course of dengue. Recent adult data indicate that the relationship between NLR and plasma leakage is strongly dependent on the day of illness; a single admission value showed only modest discriminative performance, whereas selected early-illness-day measurements performed better (Buonacera et al., 2022; Ban et al., 2026). The absence of serial sampling and the concentration of measurements within the first seven days of symptoms may therefore have obscured temporal patterns in the present cohort. These findings support interpreting NLR and SII as exploratory composite measurements rather than stable indicators of dengue severity. Previous studies have shown that NLR may vary throughout the course of dengue without consistently discriminating clinical severity, whereas the relationship between SII and dengue severity may be non-linear and influenced by host-related factors (Böer et al., 2024; Amumpuni et al., 2025).

PNI was significantly lower in women, but this result should not be interpreted as evidence of malnutrition; PNI is calculated from serum albumin and the absolute peripheral lymphocyte count, providing a composite measure of inflammatory and immunonutritional status. Although the PNI has not been widely validated in dengue, hypoalbuminemia has recently been associated with severe dengue and adverse outcomes, supporting the exploration of albumin-based composite indices in this population (Onodera et al., 1984; Shabil et al., 2025). Consequently, the observed sex-based difference is mathematically related to the lower albumin concentration and lower lymphocyte count observed in women. In acute infectious diseases, albumin levels are influenced by inflammation, the redistribution of hepatic protein synthesis, intravascular volume, and capillary permeability. Professional nutrition guidelines caution that serum albumin should not be used in isolation as a surrogate marker for total body protein or nutritional status (Evans et al., 2021). Therefore, it is more accurate to describe the lower PNI in women as a difference in a composite index of albumin and lymphocytes. To determine its nutritional relevance, it would be necessary to evaluate it alongside dietary intake, weight change, body composition or muscle mass, functional measures, and validated malnutrition criteria, such as Global Leadership Initiative on Malnutrition (GLIM) or the Academy of Nutrition and Dietetics and the American Society for Parenteral and Enteral Nutrition (ASPEN) framework (Onodera et al., 1984; White et al., 2012; Cederholm et al., 2019; Evans et al., 2021). Prospective dengue cohorts would subsequently be required to derive and externally validate disease-specific PNI cutoffs. Other differences observed in laboratory parameters warrant special attention. The higher hemoglobin and hematocrit concentrations in men were likely influenced by sex-specific baseline physiology; therefore, they cannot be interpreted as evidence of greater hemoconcentration without prior or serial measurements. Furthermore, serum creatinine and eGFR are mathematically related, and commonly used eGFR equations incorporate sex-specific parameters. In cases of acute illness, creatinine may not be at a steady state, which limits the interpretation of creatinine-based eGFR as an instantaneous measure of renal perfusion (Grams et al., 2023). The observed differences in creatinine and eGFR should not be interpreted as evidence that women had better renal perfusion or that men had dengue-associated renal dysfunction.

This study presents several strengths. All inflammatory and immunonutritional indices were calculated from individual measurements obtained from a single blood sample; the cohort showed a balanced sex distribution; and the study evaluated bedside-applicable indices derived from routinely available vital signs. The sex-stratified analysis addresses a clinically relevant source of physiological variation that has received little attention in studies on dengue in adults. However, the findings must be interpreted in light of important limitations. The cross-sectional design precluded the assessment of temporal changes, causal relationships, and predictive capacity. Dengue severity, objectively documented plasma leakage, treatment requirements, intensive care admission, and clinical outcomes were not included as reference outcomes. The timing of vital sign measurements in relation to the administration of fluids, antipyretics, analgesics, and other treatments was not fully standardized. The sample originated from a single regional setting, and its size was determined by the number of confirmed cases identified during the study period rather than by a formal *a priori* sample-size calculation; estimates for infrequent manifestations were imprecise, as reflected by wide confidence intervals. The absence of complete screening and non-enrollment data also limits assessment of potential selection bias. The numerous secondary comparisons increased the likelihood of chance findings, particularly regarding isolated clinical and biochemical differences. Potential confounding factors, such as the day of illness, temperature, comorbidities, medications, and baseline cardiovascular physiology, were not addressed in the multivariate analysis. Finally, diagnostic testing was performed by the Oaxaca State Public Health Laboratory as part of routine epidemiological surveillance, although the investigators had access to the official qualitative results and reported serotype information, raw RT-qPCR amplification files, cycle-threshold values, detailed analytical records, and other internal laboratory metadata were not available under the laboratory’s institutional data-governance and confidentiality policies. Consequently, independent verification of the molecular results, viral-load analyses, and additional classification based on raw assay parameters could not be performed; rapid NS1, IgM, and IgG patterns were therefore interpreted only as complementary descriptive findings and were not used to infer primary versus secondary infection or disease phase. Prospective studies should obtain serial measurements of the SI and MSI indices throughout the febrile, critical, and recovery phases, as defined by defervescence and clinical criteria. These indices should be evaluated against pre-established outcomes, such as the WHO classification of severe dengue, ultrasound-confirmed plasma leakage, narrowed pulse pressure, elevated lactate, the need for fluid resuscitation, admission to intensive care, and mortality. Such studies should compare discrimination capacity, calibration, and thresholds for clinical utility, and assess whether age- or sex-specific interpretation improves performance.

## Conclusions

In this cross-sectional study of adult patients with dengue from Oaxaca, Mexico, the median Shock Index was close to the prespecified alert threshold, and both SI and MSI were higher in women than in men. These differences coincided with a higher heart rate, whereas systolic, diastolic, pulse, and mean arterial pressures did not differ significantly between the sexes. NLR and SII were comparable between groups, whereas PNI was lower in women; however, this difference in the composite should not be interpreted as direct evidence of impaired nutritional status. Because the study did not include longitudinal measurements, a reference standard for circulatory compromise, severity stratification, or clinical outcomes, the findings do not establish diagnostic or prognostic utility. They support prospective evaluation of serial SI and MSI measurements against objectively defined dengue severity and circulatory outcomes, accounting for age-, sex-, treatment-, and illness-day-related variation.

## Data Availability

The article includes summary data supporting the study's findings. Readers can direct further inquiries to the corresponding authors. Raw RT-qPCR amplification files and other internal laboratory records were not available to the authors, as described in the Methods.
